# One Health Approach to Leptospirosis: Dogs as Environmental Sentinels for Identification and Monitoring of Human Risk Areas in Southern Brazil

**DOI:** 10.3390/tropicalmed8090435

**Published:** 2023-09-06

**Authors:** Natacha Sohn-Hausner, Louise Bach Kmetiuk, Evelyn Cristine da Silva, Helio Langoni, Alexander Welker Biondo

**Affiliations:** 1 Department of Cell and Molecular Biology, Federal University of Paraná, Curitiba 80035-050, PR, Brazil; 2 Department of Veterinary Hygiene and Public Health, Sao Paulo State University, Botucatu 18618-681, SP, Brazil

**Keywords:** health policy, vulnerability, risk mapping, sentinel dog, vulnerable populations, zoonoses

## Abstract

Leptospirosis has been a neglected, widespread and reemerging zoonotic disease of global importance. The One Health holistic approach combining human, animal and environmental health has been important for control and prevention of zoonotic disease. An urban municipality in Southern Brazil with a high prevalence of leptospirosis was selected by convenience, with asymptomatic human and canine individuals serologically tested using MAT against 30 *Leptospira* spp. serovars. Epidemiological questionnaires were assessed along with the historical national database, with associated risk factors to dog and human seropositivity analyzed using multiple logistic regression. The spatial distribution of retrospective human leptospirosis cases was analyzed using a Kernel map and overlapped dog seropositivity and historical flooding areas, demonstrating that domestic and asymptomatic dogs may be important environmental sentinels for leptospirosis in such urban areas, even in the absence of human seropositivity and low canine seropositivity. Associated risk factors for leptospirosis in dogs and humans according to multiple regression included healthy dogs (*p* = 0.02, odds ratio—OR = 0.17, confidence interval of 95%CI 0.03–0.71) with indoor access (*p* = 0.059, OR = 5.12, 95%CI 1.10–37.7) and human males (*p* = 0.042, OR = 2.44, 95%CI 1.08–6.11) with presenting calf pain (*p* = 0.005, OR = 3.14, 95%CI 1.46–7. 14), jaundice (*p* < 0.001, OR = 4.61, 95%CI 2.11–10.10) and renal failure (*p* = 0.008, OR = 4.49, 95%CI 1.49–13.76). The greater the rain precipitation (above 3 mm of average annual precipitation), the higher the number of leptospirosis cases in humans. In conclusion, dog active serosurvey and rain precipitation should be systematically reported and plotted altogether as a basis for the early detection and monitoring of human risk areas. Such findings may serve as a basis for public health policies in Brazil and other endemic countries worldwide.

## 1. Introduction

Leptospirosis has been a neglected and reemerging disease of global importance for public health with high morbidity and mortality in humans and animals [1] and is considered the most widespread zoonotic disease worldwide [2]. The disease is caused by bacteria of the genus *Leptospira* (order Spirochaetales, family Leptospiraceae), of which 64 species have been recognized and divided into two main clades: “Saprophytes” containing noninfectious species isolated in a natural environment and “Pathogens” including all responsible species for human and animal infections, along with environmental species with unproven virulence [3]. In this last group are the species (*L. interrogans*, *L. kirschneri*, *L. noguchii*, *L. santarosai*, *L.mayottensis*, *L. borgpetersenii*, *L. alexanderi* and *L. weilii*) that have been mostly associated with severe human infections [3] while *L. interrogans*, *L. kirschneri*, *L. noguchii*, *L. santarosai*, *L. borgpetersenii* and *L. weilii* have been reportedly found in dogs from Brazil [4]. Optimal conditions for *Leptospira* growth have been reported at pH 6.7 to 8.0 in river water and nearby moist soils [5], while a high salt concentration may not be suitable for the long-term survival of pathogenic *Leptospira* [6]. A recent systematic review has shown that *Leptospira* spp. may survive in the environment and keep virulence for more than 20 days in water and 40 days in soil, varying among species and strains [6]. Survival and virulence of *Leptospira interrogans* for about 20 months have been observed in bottled mineral water [7] and for 6 months in water-saturated soil [8]. As no definitive proof has been obtained by open-field studies, water and soil may be repeatedly contaminated by animals and the surrounding environment. In addition, *Leptospira* environmental survival may be favored by multispecies natural biofilm formation and protection, with virulent *Leptospira* interactions with complex environmental microbiota [6]. Regardless, *Leptospira* infection mostly occurs through the urine of infected rats [9] and other domestic [10] and wild [11] animals, favored in tropical regions [1] due to exposure to contaminated flooding water [9]. As human cases generally occur after heavy rain, rainfall water may resuspend and spread leptospires together with soil particles to infect humans or animals through body wounds in the skin, mucous membranes and conjunctiva [6]. Despite surviving in contaminated urine and water in the environment, pathogenic leptospires do not multiply in such conditions [6]. As human-to-human transmission has been rarely reported [12], the One Health approach has been mandatory to better understanding the animal and environmental role in the maintenance of *Leptospira* spp., as well as indicating risk areas for the monitoring and prevention of new human and animal cases.

Leptospirosis has been considered an underdiagnosed infection mostly due to asymptomatic and nonpathognomonic initial symptoms, similar to other febrile-hemorrhagic-icteric illnesses such as the flu, hepatitis, Dengue fever and hantaviruses, making the clinical diagnosis a challenge and leading to case underreporting [12,13]. Thus, despite the estimative annual worldwide incidence of 1.03 million cases, 58,900 deaths and losses of 2.9 million of disability-adjusted life years, these numbers should be actually even higher [13]. The highest morbidity regions include South and Southeast Asia, Oceania, the Caribbean, sub-Saharan Africa and Latin America, mainly due to the high humidity and presence of broadleaf forests, with the highest reports of leptospirosis outbreaks (32.1%; 102/318) [12]. The estimated annual morbidity rates in the Andean and South Latin America have ranged from 1.43 to 39.8 per 100,000 population; Brazil has accounted for 40.2% of reported cases with nearly half of the Latin American population [12]. In Brazil, the notification of leptospirosis cases became mandatory in 2000 and nationwide surveillance has been conducted by the Brazilian Ministry of Health, with an annual average of 3846 cases, an incidence of 1.9 per 100,000 inhabitants and an 8.9% lethality rate, with a steady average over the past 20 years [14]. Investigations herein were conducted first at the local level (municipality), based on case notifications, then on state and national levels, based on the Ministry of Health dataset of notified cases, publishing standards and surveillance recommendations [15]. The current preventive strategy for human leptospirosis has been focused on disease historical epidemiology and postoccurrence of natural disasters, such as floods and inundations, with rapid public information and early identification of suspicious cases [14].

Leptospirosis has been historically endemic throughout the Brazilian territory, with a higher incidence of human cases in the southern, southeastern and northern regions, particularly in urban areas of major cities [4]. In addition, a systematic review of dog leptospirosis in Brazil has shown overlapping with flooding areas, indicating the need for local scale studies to confirm dogs as environmental sentinels in urban areas [4], as dog the role of dogs in the *Leptospira* cycle remains to be fully established [4,10,16]. Dogs have been considered as accidental hosts for most serovars except Canicola, for which dogs have been reportedly considered as maintenance hosts [17]. Among the 36 known *Leptospira* spp. serovars, the most prevalent with variable distribution across Brazilian states are Autumnalis, Canicola, Copenhageni, Grippotyphosa and Icterohaemorrhagiae [18]. Icterohaemorrhagiae and Copenhageni have been frequently associated with severe human cases in Brazil, highlighting the role of dogs as reservoirs of these serovars in urban areas [19]. Canine leptospirosis has been considered a public health concern due to pathogenicity, an extended shedding period [19] and potential dog–human transmission [19]. Sharing the same household environment, dogs have been of high importance in terms of leptospirosis, including its investigation, control and prevention [19,20], particularly unvaccinated dogs, commonly found in low-income populations [21].

The One Health approach combining human, animal and environmental health in a holistic approach has been important for zoonotic disease control and prevention [22]. Recently applied to leptospirosis, spatial human–dog analysis along with rainfall and flooding have provided a better approach to household exposure, cross-infection, associated risk factors and mapping of risk areas [18]. Despite this study describing the human–dog–environment dynamics of leptospirosis in Brazil over a 20-year period, the role of dogs at the local level, particularly in urban areas, remains to be fully established. Accordingly, the present study has aimed to identify local *Leptospira* spp. serovars in owners and their dogs, the associated risk factors, the association with rainfall and flooding areas and an assessment of owned asymptomatic dogs as potential environmental sentinels for risk areas of human leptospirosis.

## 2. Materials and Methods

### 2.1. Ethics Statement

The present study was approved by protocol number 34934220.4.0000.0102/2020 of the National Research Ethics Commission, Brazil, and protocol number 078/2019 of the Ethics Committee on the Use of Animals at the Federal University of Paraná.

### 2.2. Study Area

**Figure 1:**
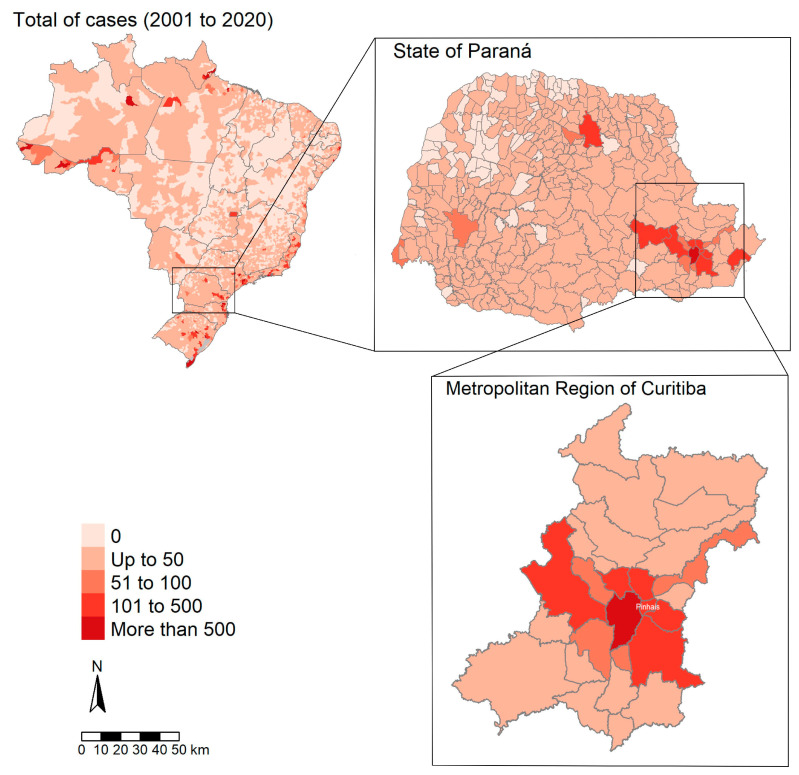
Map of Brazil with compilation of the history (2001 to 2020) of human leptospirosis cases [18], emphasizing the area of Paraná state, metropolitan region of Curitiba and the city of Pinhais, the local scale analysis of the present study.

The study herein occurred in the municipality of Pinhais (25°25′57″ S and 49°11′35″ W, 930 m, 60.75 km^2^), with 129,445 inhabitants and 100% urban area, located within the metropolitan area of Curitiba, Paraná state capital, southern Brazil (Figure 1). The chosen area is the eighth largest Brazilian metropolitan, concentrating around a third (30.86%) of the state’s population with 3,731,769 inhabitants, and also the second-largest metropolitan region in extension nationwide at 16,581.21 km^2^ [23]. The municipality has a Cfb classification on the Köppen climate, with 1550 mm annual rainfall and a 17 °C average annual temperature [24,25]. It is divided into 15 neighborhoods and subdivided into four hydrographic regions (Iraí river, do Meio river, Palmital river and Atuba river) with different environmental and population characteristics [26].

### 2.3. Sample and Data Collection for Analysis and Mapping of Human and Dog Leptospirosis Cases

Blood samples and epidemiological questionnaires in the present study were collected by a multidisciplinary taskforce team in campaigns during 23 field days (April and September 2019, January to March and November 2020), as part of another study on the ecology of ticks and diseases transmitted by ticks and based on complaints of tick infestation in households received by the Health Department of Pinhais. All addresses were georeferenced, gathered, analyzed and used for risk map construction of leptospirosis in the municipality of Pinhais. All statistical analyses were performed in an environment R 4.0.4 [27] with a significance level of 5%.

#### 2.3.1. Questionnaires

All participants were volunteers and signed an informed, explained consent before questionnaire application and blood samplings. Epidemiological questionnaires were voluntarily taken by asymptomatic residents and about their dogs to determine the risk factors for *Leptospira* spp. infection. Questionnaires included closed questions on different variables that may be associated with owner and dog exposure to leptospirosis, such as personal sanitary habits, sanitary access, animal habits and management and environmental management.

#### 2.3.2. Human and Dog Blood Samples

Whole blood samples were collected in sterile tubes containing separating gel using venipuncture of the median cubital vein in humans, performed by a certified nurse, and of the cephalic or jugular vein in dogs, performed by a certified veterinarian. After collection, samples were centrifuged at 1500 rpm for 10 min, the serum was obtained, aliquoted and placed into microtubes, and stored at −70 °C until the serological analysis.

#### 2.3.3. History of Leptospirosis Cases Reported in the City of Pinhais

The historical records (2007 to 2010) of compulsory notifications made for patients living in Pinhais with suspected leptospirosis (CID-10 A27.9) were obtained through the Notifiable Diseases Information System—SINAN [28] of the Health Department of Pinhais. This period was selected due to SINAN availability of microdata (information contained in the investigation questionnaires) only for these years. The informed addresses in the notifications were georeferenced and used to build risk maps for diseases in the city (positive and negative cases).

#### 2.3.4. History Precipitation for Pinhais City

The historical data (2010 to 2020) of the average annual precipitation for the municipality was officially requested from and made available by the Paraná Environmental Technology and Monitoring System—SIMEPAR. The obtained information was plotted on a graph and compared, per year, with the number of confirmed cases of leptospirosis in the municipality.

#### 2.3.5. Spatial Data

Cartographic bases of shapefiles (.shp, .shx, .dbf and .prj) and/or Keyhole Markup Language (.kml) files and other geographic information, along with a history of flooding quotas and the division of neighborhood and municipality borders were obtained from the Urban Planning Department of the Pinhais Urban Planning Department [29] and used to build risk maps.

### 2.4. Serological Analysis 

The microscopic agglutination test (MAT) was applied to 21 serogroups of *Leptospira* spp., which comprised a collection of 30 different serovars. All blood sera were analyzed for this series of serovars, including Andamana, Australis, Autumnalis, Bataviae, Bratislava, Bovis, Canicola, Castellonis, Copenhageni, Cynopteri, CTG, Djasiman, Grippotyphosa, Guaricura, Hardjo, Hebdomadis, Icterohaemorraghiae, Javanica, Minis, Nupezo-01, Patoc, Panama, Pomona, Pyrogenes, Prajtino, Sentot, Shermani, Tarassovi, Whitcombi and Wolffi. The reference strains used in the study were maintained at the Zoonoses Research Center (NUPEZO) of the Faculty of Veterinary Medicine and Animal Science of the São Paulo State University (UNESP)—Campus Botucatu, São Paulo, Brazil. All samples submitted to serological tests were analyzed at dilutions 1:100 to 1:3200, considering the highest positive dilution the correspondent serum titer [30,31,32]. The 1:100 dilution was used as a cutoff point as previously recommended [26,30]. Serum samples were considered positive when at least 50% of the leptospiral bacterial suspensions agglutinated read under a dark field optical microscope. Negative controls were used for each serovar in all serological assays.

### 2.5. Statistical and Spatial Analysis

The results were divided into SINAN data and questionnaire data for descriptive and inference analyses. Initially, data were descriptively analyzed using simple (n) and relative (%) frequency estimates in the two data sets of all variables. Then, association with case confirmation [33] or with positive results (questionnaire data) was assessed using the Chi-square test and odds ratio (OR) estimate and a 95% confidence interval. Multiple logistic regression models were applied to obtain the case profiles, with variables with *p* < 0.20 selected to start the models in the multiple modeling. The used input and output method of variables was stepwise starting from the most to the simplest complex model. The criteria used in the final model were >10% change in OR, improvement in the accuracy of the 95% CI, degrees of freedom, statistical significance and the model’s AIC adjustment. The significance level was considered to be 5%. Spatial analyses were applied to the georeferencing addresses (locations) of complaints and reported cases (SINAN); thematic maps and cluster analysis (kernel density) were also constructed. All analyses were performed in the R 4.0.4 environment [34].

## 3. Results

### 3.1. Prospective MAT Tests of Human and Dog Samples

Overall, no human (0/135) and 10/133 (7.52%) dog samples were reactive for *Leptospira* spp. using the microscopic agglutination technique (MAT). There were agglutination results of dog samples including Icterohaemorragiae (n = 6, with titers of 100 to 400), Pyrogenes (n = 4, with titers of 100 and 200), Copenhageni (n = 4, with titers of 100 and 200), Canicola (n = 3, with titers of 100 and 200) and Autumnalis (n = 2, with titers of 100). Three dogs were reactive for more than one serovar (Table 1).

### 3.2. Regression Model of Questionnaire Data

As no seropositive human sample was found in the prospective serological investigation in the municipality of Pinhais, only the information contained in the Notifiable Diseases Information System—SINAN investigation questionnaires were used for analysis. Risk factors for dogs were based on the information from the epidemiological questionnaires given to dog owners.

The analyses of human variables showed males (*p* = 0.042, odds ratio—OR = 2.44, confidence interval of 95%—95%CI 1.08–6.11) as a risk factor for leptospirosis in humans, while the model indicated the presence of clinical symptoms including “calf pain” (*p* = 0.005, OR = 3.14, 95%CI 1.46–7.14), “jaundice” (*p* < 0.001, OR = 4.61, 95%CI 2.11–10.10) and “renal failure” (*p* = 0.008, OR = 4.49, 95%CI 1.49–13.76) as associated risk factors for human leptospirosis (Table 2, Supplementary Tables S1–S3). The dog model showed the absence of ticks (*p* = 0.021, OR = 0.17, 95%CI 0.03–0.71) and dog indoor access (“enter the house” *p* = 0.059, OR = 5.12, 95%CI 1.10–37.7) as risk factors for leptospirosis (Table 3, Supplementary Tables S4 and S5).

### 3.3. Relationship between Human Leptospirosis Cases and MAT in Dogs and Average Annual Precipitation and Flooding Areas

**Figure 2:**
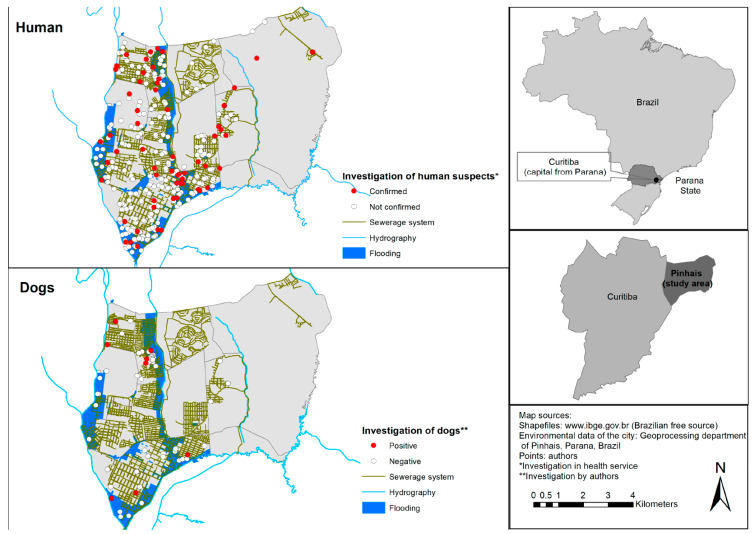
Map of the geographical locations of (seropositive and seronegative) human and dog cases in Pinhais, historic flooding areas and chart with number of positive human cases notified on SINAN per year (2007 to 2020) and average annual precipitation (2010–2020).

The location of notifications for investigation of human (positive and negative) cases and results (seropositive and seronegative) of dog MAT, both obtained from the georeferencing of the patient’s residence and sample collection addresses (respectively), were plotted on a map (Figure 2) with the occurrence of flooding areas, sewage system and hydrography of Pinhais. Although positive spot cases within the flooding area (up to 2 squares distance) showed a clustering tendency to nearby flooding areas, counting was visually performed and may not represent a true association (Figure 2).

**Figure 3:**
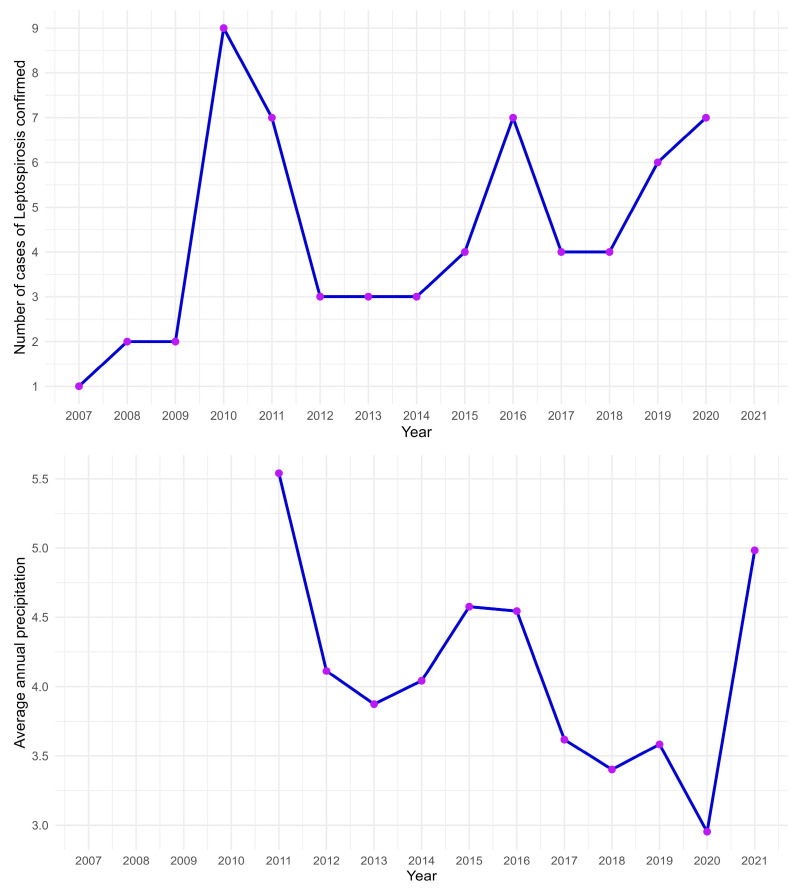
Number of cases of leptospirosis confirmed for the municipality of Pinhais, 2007–2020 and average annual precipitation for municipality of Pinhais, 2010–2020.

**Figure 4:**
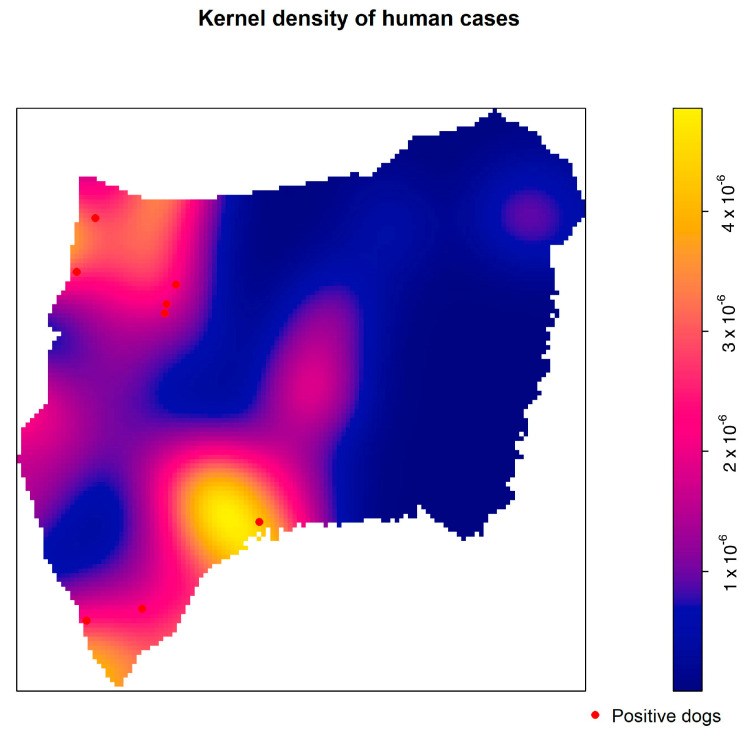
Map of the kernel density of human leptospirosis cases (Pinhais, 2007 to 2020) with the location of seropositive dogs in the serological research of *Leptospira* spp. (Pinhais, 2019 and 2020).

Graphs of leptospirosis cases per year presented a positive relationship with the average annual rainfall in Pinhais (Figure 3 and Figure 4).

### 3.4. Dogs as Environmental Sentinels for Leptospira spp.

Superimposition of seropositive dogs with the kernel density of positive human cases (Figure 4) showed spatial proximity, demonstrating that dogs, in Pinhais, were environmental sentinels for *Leptospira* spp. At the time of the survey.

## 4. Discussion

The World Health Organization (WHO) has recommended an increase in leptospirosis surveillance, focused on the One Health Initiative to better determine global losses, development of surveillance methods and establish effective disease control and prevention measures, in small and defined populations (such as dogs) as a highly effective control measure [22]. The present study has indicated that, in the absence of seropositive human samples, seropositive dogs have shown to be important environmental sentinels for leptospirosis, even with relatively low prevalence (Table 1). Not surprisingly, seropositive asymptomatic dogs herein were mostly found in or close to areas where historical floods occurred in the municipality (Figure 4). Previous studies have also shown that dog exposure to leptospirosis has been positively correlated with human infection, indicating that dogs may play an important role as disease sentinels [30,31,32]. In addition, spatial and simultaneous comparison of owner and dog serology has been reported, analyzing cross-infection risks, common domestic environmental exposure and spatially verifying dogs as environmental sentinels for *Leptospira* spp. in urban areas [10]. Dogs have also been recently indicated as sentinels in a One Health approach to leptospirosis in Brazil in a broader nationwide scale analysis [18,35]. It is important to emphasize that dogs in the present study have been considered environmental sentinels for human leptospirosis as indicators for risk areas of human disease. As no human sample herein was seropositive for leptospirosis in the city prospective serosurvey, direct comparison failed to show a discernible dog–human correlation. However, retrospective city results of cases fulfilled the role of dogs as sentinels for risk areas of human leptospirosis.

Although human leptospirosis cases have been reported by SINAN in the municipality of Pinhais (Figure 2), no seropositive human sample was detected by MAT in the present study. Despite a higher incidence of leptospirosis cases in men [36], the convenience sampling of 67.4% female participants herein may have not influenced the results as overall leptospirosis incidence in the Parana state was 2.98 per 100,000 habitants, below human samplings performed in the present study. Nonetheless, as the study herein was performed in high-risk areas, including historical flooding areas, active surveys could have provided at least seropositive asymptomatic cases.

Despite restricted regional access and a low sample size, the study herein aimed to refine the survey on a local scale, with 10/133 (7.52) seropositive dogs for *Leptospira interrogans*, lower than a previous same-city report with 33/228 (14.4%) dogs in 2009 and 35/90 (38.9%) in 2010 (14.4 to 38.9%, mean 26.6%) [4,36] and within the range from 35/378 (9.3%) to 36/189 (19.0%) in a monitoring study (0 to 27.8%, average 13.7%) in Curitiba, the neighboring state capital and the eighth largest Brazilian city [37,38,39,40,41]. In addition, results herein were within the range of the northern Paraná state (0 to 46.7%, average 18.6%) with 35/175 (20.0%) in 2011, 41/335 (12%) in 2006–2008, 51/236 (21.6%) in 2004–2012 and 155/729 (21.3%) in 2015–2016 [10,37,38,39,40,41,42,43,44,45,46,47]. Such fluctuation of anti-*Leptospira* spp. antibody prevalence in dogs may be a consequence of several factors including the rodent population and environmental conditions such as temperature, rainfall and flooding [47,48]. As expected, the municipality of Pinhais presented the highest dog seropositivity (38.9%) in 2010 [4], the highest number of human leptospirosis cases reported by SINAN (Figure 3), along with a major city flooding affecting around 30,000 habitants [49,50,51]. 

The *Leptospira interrogans* serovars found in dogs herein (Copenhageni, Pyrogenes, Icterohaemorragiae, Canicola and Autumnalis) have been frequently found in Brazilian dogs nationwide [18,52] and, with the exception of Pyrogenes, the most prevalent in animals of the Americas [53] also infecting humans [10,53,54]. The serogroup Icterohaemorrhagiae (serovars Icterohaemorrhagiae and Copenhageni) in dogs has been likely associated with infestation and contact with *Rattus norvegicus*, the main host species of this serogroup [55]. The serovar Canicola, recognized as adapted to dogs and the main serovar reported in Europe and South America [56,57,58], confirmed the role of asymptomatic dogs in maintaining this serovar [59,60]. While the serovar Pyrogenes has been recurrent [61] and considered accidental in Brazilian dogs [3,62], Autumnalis has been identified as the main infective serogroup in stray and asymptomatic dogs in Brazil, Latin American islands (Barbados, Saint Kitts and Trinidad) and Japan, with potential zoonotic transmission [63,64,65]. The identification of serovar occurrence and control of rodent and stray dog populations have been critical for the effective reduction of leptospirosis in dogs and, consequently, the risk for humans [66]. In addition, the overlapping of circulating serovars may indicate similar human–dog exposure to associated risk factors [67]. Finally, commercial dog vaccines have relied on inactivated leptospires of the most prevalent serovars, with no human vaccine available to date in Brazil [68]. In such a scenario, the identification of circulating *Leptospira* spp. serovars in dogs may contribute to a better understanding of the zoonotic risk in a given area since dogs, particularly those with outdoor access, may be infected by other animals (such as rats) and contaminated soil and water, shedding leptospires through urine and infecting humans.

Although no human seropositivity was found herein, associated risk factors included “males” (*p* = 0.042), “jaundice” (*p* < 0.001), “calf pain” (*p* = 0.005) and “renal failure” (*p* = 0.008) (Table 2). Despite not being statistically significant, “myalgia” (*p* = 0.075), “presence of garbage” (*p* = 0.587) and “contact with flood water or mud” (*p* = 0.102) were also associated with positive human cases in the final model (S2). In dogs, the final multiple logistic regression model showed dogs “without ectoparasites” (*p* = 0.021) and “entering the residence” (*p* = 0.059) as more likely seropositive to *Leptospira* spp. infection (Table 3). Although cohabitation may have facilitated human infection, direct dog transmission was considered uncommon [10,69] and the results may indicate similar human–dog exposure to shedding leptospires from the urine of infected rats. Thus, the absence of seropositive human samples in the current study has not diminished the importance of such findings, emphasizing that negative results in humans may still be valuable in understanding the dynamics of leptospirosis transmission in the study area.

As they are more exposed to environmental infection sources than humans, dogs may indicate the epidemiological links between environmental sources and humans [30,66]. The most frequent environmental risk factors for humans have been related to exposure to contaminated water, mainly flooding and high rainfall, and exposure related to poor sanitation (sewage and garbage) and the presence of rodents and dogs [70]. Not surprisingly, as the probability of infection may reach 68% higher in dogs with environmental water sources [66], human cases with seropositive dogs herein were associated with flooding areas, indicating risk hotspots for leptospirosis on a local scale (Figure 4). Thus, in addition to the spatial overlapping of human–dog leptospirosis cases in Brazil [10,18], the present study has shown such a pattern and role of dogs as potential environmental sentinels for leptospirosis in urban areas within the same city.

As limitations, the present leptospirosis study was based on the SINAN passive surveillance system, which may be underreported, undiagnosed or misdiagnosed [71,72]. In Brazil, disease notification has relied on health professionals correctly including cases in the national system [73], which may be impaired by lack of computer access, internet connection and unqualified or untrained professionals. In addition, despite MAT high specificity being the “gold standard method” for detecting leptospirosis, this test requires a large panel of serovars and laborious maintenance of correspondent live strains. As the *Leptospira* serovar panel has been built on historical prevalence, recent or new species and serovars may not be identified, despite causing human and animal disease [73]. Finally, due to the nonspecific symptoms of leptospirosis, individuals with mild symptoms may not seek health care and others may be often confused as being associated with other febrile diseases such as dengue and malaria [73,74].

In summary, despite seroprevalence being absent in humans and very low in dogs, the main goal of this study was to determine the role of dogs as environmental sentinels for human risk of leptospirosis, even with low dog sampling due to convenience and low prevalence. The human retrospective study of leptospirosis cases in this region was used to better explain the role and the human–dog overlapping of seropositive cases. In addition, active serosurvey in dogs may provide early diagnosis and appropriate treatment and disease control, preventing human infections.

## Figures and Tables

**Table 1:** Results of MAT serological tests of asymptomatic dogs from Pinhais-PR (2019 and 2020).

Variable		N	%	CI 95%
*Leptospira* spp.	Seropositive	10	7.52	4.14–13.29
	Seronegative	123	92.48	86.71–95.86
Serogroup	Autumnalis	2	1.5	0.41–5.32
	Canicola	3	2.26	0.77–6.42
	Icterohaemorragiae	6	4.51	2.08–9.49
	Pyrogenes	4	3.01	1.18–7.48
Serovar (title)	Copenhageni (100)	3	2.26	0.77–6.42
	Copenhageni (200)	1	0.75	0.13–4.14
	Icterohaemorragiae (100)	2	1.5	0.41–5.32
	Icterohaemorragiae (400)	1	0.75	0.13–4.14
	Pyrogenes (100)	3	2.26	0.77–6.42
	Pyrogenes (200)	1	0.75	0.13–4.14
	Autumnalis (100)	2	1.5	0.41–5.32
	Canicola (100)	2	1.5	0.41–5.32
	Canicola (200)	1	0.75	0.13–4.14

**Table 2:** Results of final multiple logistic regression model with the selected independent variables (stepwise) and their respective odds ratio (OR), confidence interval (IC) and *p*-value of the SINAN investigation questionnaires for human leptospirosis (N = 378), Pinhais-PR, 2007 to 2020.

Final Model			
Independent Variables	OR	IC	*p*-Value
(Intercept)	0.01	0.00–0.03	<0.001
Sex: male	2.44	1.08–6.11	0.042
Risk flood water or mud: yes	1.85	0.89–3.89	0.102
Trash/debris risk: yes	1.24	0.55–2.70	0.587
Myalgia: yes	2.92	0.99–11.07	0.075
Calf pain: yes	3.14	1.46–7.14	0.005
Jaundice: yes	4.61	2.11–10.10	<0.001
Kidney failure: yes	4.49	1.49–13.76	0.008
Evolution: death and other causes	0.46	0.02–3.32	0.503

**Table 3:** Results of the final multiple logistic regression model with the selected independent variables (stepwise) and their respective odds ratio (OR), confidence interval (IC) and *p*-value of the epidemiological questionnaires for dog leptospirosis (Pinhais-PR, 2007 to 2020).

Final Model			
Independent Variables	OR	IC	*p*-Value
(Intercept)	0.11	0.02–0.38	0.03
No tick presence	0.17	0.03–0.71	0.021
Enters home: yes	5.12	1.10–37.71	0.059

## Data Availability

Not applicable.

## Supplementary Materials

The following supporting information can be downloaded at: https://www.mdpi.com/article/10.3390/tropicalmed8090435/s1, Table S1. Associated risk factors according to confirmed and discarded cases from data contained in the Sinan Investigation Questionnaires (2007–2020) for human residents of Pinhais, Paraná state, southern Brazil (N = 403), by uni- and multivariate statistical analysis. Table S2. Multiple logistic regression models by the stepwise method of input and output of Human variables. Table S3. Number of co-variables from Human Models and their respective AIC. Table S4. Associated risk factors for anti-Leptospira agglutinins (2019–2020) in dog population of of Pinhais. Paraná state. southern Brazil (N = 133). by uni- and multivariate statistical analysis. Table S5. Multiple logistic regression models by the stepwise method of input and output of dog variables.
