# Supplementary material for: One Health Approach to Leptospirosis: Dogs as Environmental Sentinels for Identification and Monitoring of Human Risk Areas in Southern Brazil
Source: Trop Med Infect Dis. 2023 Sep 6;8(9):435. doi: 10.3390/tropicalmed8090435 (PMC10534403; doi:10.3390/tropicalmed8090435)
Supplement: Supplementary file 1 [file tropicalmed-08-00435-s001.zip › tropicalmed-2525776-supplementary.pdf]

Tropical Medicine and Infectious Disease

MDPI

Supplementary Materials of One Health Approach to Leptospirosis: Dogs as Environmental Sentinels for Identification and Monitoring of Human Risk Areas in Southern Brazil

Table S1. Associated risk factors according to confirmed and discarded cases from data contained in the Sinan Investigation Questionnaires (2007-2020) for human residents of Pinhais, Paraná state, southern Brazil (N = 403), by uni- and multivariate statistical analysis.

Variable		Confirmed Cases		Discarded Cases		Total	%	OR	IC	*p*-value
		N	% lin.	N	% lin.					
**Sex**	Female	9	6.62	127	93.38	136	100	Ref	-	-
	Male	53	21.9	189	78.1	242	100	3.96	1.89-8.31	<0.001
Ethinicity	White	45	16.92	221	83.08	266	100	Ref	-	-
	Yellow	0	0	5	100	5	100	-	-	0.314
	Brown	10	20.83	38	79.17	48	100	1.29	0.6-2.78	0.511
	Black	2	8.7	21	91.3	23	100	0.46	0.11-2.07	0.305
	No Filling	5	13.89	31	86.11	36	100	0.79	0.29-2.15	0.646
Education	Illiterate *	0	0	5	100	5	100	0	-	0.206
	Elementary	24	24.49	74	75.51	98	100	Ref	-	-
	High school	8	12.70	55	87.30	63	100	0.45	0.18-1.07	0.067
	Higher education	1	11.11	8	88.89	9	100	0.39	0.04-3.24	0.364
	Not applicable	0	0	19	100	19	100	0	-	0.015
	No Filling	29	15.76	155	84.24%	184	100	0.58	0.31-1.06	0.074
**Flood water or mud**	No	24	11.27	189	88.73	213	100	Ref	-	-
	Yes	33	23.24	109	76.76	142	100	2.38	1.34-4.24	0.003
	No Filling	5	21.74	18	78.26	23	100	2.19	0.74-6.43	0.146
Animal husbandry	No	47	15.36	259	84.64	306	100	Ref	-	-

	Yes	9	20.45	35	79.55	44	100	1.42	0.64-3.14	0.389
	No Filling	6	21.43	22	78.57	28	100	1.5	0.58-3.9	0.4
	No	55	16.47	279	83.53	334	100	Ref	-	-
Water tank	Yes	1	6.67	14	93.33	15	100	0.36	0.05-2.81	0.312
	No Filling	6	20.69	23	79.31	29	100	1.32	0.51-3.4	0.56
	No	47	15.61	254	84.39	301	100	Ref	-	-
Pit, grease trap or sewer	Yes	9	18.37	40	81.63	49	100	1.22	0.55-2.67	0.626
	No Filling	6	21.43	22	78.57	28	100	1.47	0.57-3.83	0.423
	No	28	15.38	154	84.62	182	100	Ref	-	-
Place with rodents	Yes	28	16.67	140	83.33	168	100	1.1	0.62-1.95	0.744
	No Filling	6	21.43	22	78.57	28	100	1.5	0.56-4.03	0.419
	No	52	15.52	283	84.48	335	100	Ref	-	-
Planting/harvesting (cropping)	Yes	3	25	9	75	12	100	1.81	0.48-6.93	0.377
	No Filling	7	22.58	24	77.42	31	100	1.59	0.65-3.87	0.307
	No	37	13.36	240	86.64	277	100	Ref	-	-
**River, stream, pond or dam**	Yes	23	29.49	55	70.51	78	100	2.71	1.49-4.93	<0.001
	No Filling	2	8.7	21	91.3	23	100	0.62	0.14-2.74	0.523
	No	42	15.22	234	84.78	276	100	Ref	-	-
Contact with rodent	Yes	15	20	60	80	75	100	1.39	0.72-2.68	0.319
	No Filling	5	18.52	22	81.48	27	100	1.27	0.45-3.53	0.651
	No	55	16.08	287	83.92	342	100	Ref	-	-
Grain/food storage	Yes	1	11.11	8	88.89	9	100	0.65	0.08-5.32	0.688
	No Filling	6	22.22	21	77.78	27	100	1.49	0.58-3.86	0.408
	No	42	14.29	252	85.71	294	100	Ref	-	-
Wasteland	Yes	13	23.21	43	76.79	56	100	1.81	0.9-3.66	0.092
	No Filling	7	25	21	75	28	100	2	0.8-5	0.131
	No	38	13.97	234	86.03	272	100	Ref	-	-
**Garbage/debris**	Yes	19	23.75	61	76.25	80	100	1.92	1.03-3.56	0.037

	No Filling	5	19.23	21	80.77	26	100	1.47	0.52-4.12	0.466
	No	10	10	90	90	100	100	Ref	-	-
**Fever**	Yes	52	18.84	224	81.16	276	100	2.09	1.02-4.29	0.041
	Ignored	0	0	2	100	2	100	-	-	0.638
	No	5	5.49	86	94.51	91	100	Ref	-	-
**Myalgia**	Yes	57	20.28	224	79.72	281	100	4.38	1.7-11.29	0.001
	Ignored	0	0	6	100	6	100	-	-	0.555
	No	20	16.39	102	83.61	122	100	Ref	-	-
Headache	Yes	41	16.47	208	83.53	249	100	1.01	0.56-1.8	0.986
	Ignored	1	14.29	6	85.71	7	100	0.85	0.1-7.45	0.883
	No	29	14.22	175	85.78	204	100	Ref	-	-
Prostration	Yes	31	19.02	132	80.98	163	100	1.42	0.81-2.47	0.216
	Ignored	2	18.18	9	81.82	11	100	1.34	0.28-6.52	0.715
	No	46	14.51	271	85.49	317	100	Ref	-	-
**Conjunctival congestion**	Yes	16	29.63	38	70.37	54	100	0.48	1.28-4.81	0.006
	Ignored	0	0	7	100	7	100	-	-	0.277
	No	17	8.95	173	91.05	190	100	Ref	-	-
Calf pain	Yes	44	24.72	134	75.28	178	100	3.34	1.83-6.11	<0.001
	Ignored	1	10	9	90	10	100	1.13	0.14-9.47	<0.001
	No	26	11.5	200	88.5	226	100	Ref	-	-
**Vomit**	Yes	35	23.97	111	76.03	146	100	2.43	1.39-4.24	0.002
	Ignored	1	16.67	5	83.33	6	100	1.54	0.17-13.69	0.697
	No	41	14.49	242	85.51	283	100	Ref	-	-
Diarrhea	Yes	20	22.99	67	77.01	87	100	1.76	0.97-3.21	0.062
	Ignored	1	12.5	7	87.5	8	100	0.84	0.1-7.03	0.875
	No	28	9.89	255	90.11	283	100	Ref	-	-
**Jaundice**	Yes	34	37.78	56	62.22	90	100	5.53	3.1-9.85	<0.001
	Ignored	0	0	5	100	5	100	-	-	0.459

	No	45	13.39	291	86.61	336	100	Ref	-	-
**Renal insufficiency**	Yes	17	56.67	13	43.33	30	100	8.46	3.85-18.58	<0.001
	Ignored	0	0	12	100	12	100	-	-	0.174
	No	48	14.95	273	85.05	321	100	Ref	-	-
**Respiratory alteration**	Yes	13	26.53	36	73.47	49	100	2.05	1.02-4.15	0.042
	Ignored	1	12.5	7	87.5	8	100	-	-	0.847
	No	58	16.25	299	83.75	357	100	Ref	-	-
Cardiavascular alteration	Yes	2	22.22	7	77.78	9	100	1.47	0.3-7.27	0.642
	Ignored	2	16.67	10	83.33	12	100	1.03	0.22-4.83	0.969
	No	59	16.34	302	83.66	361	100	Ref	-	-
Lung hemorrhage	Yes	3	33.33	6	66.67	9	100	2.56	0.62-10.52	0.178
	Ignored	0	0	8	100	8	100	-	-	0.212
Other hemorrhages	No	58	16.2	300	83.8	358	100	Ref	-	-
	Yes	4	33.33	8	66.67	12	100	2.59	0.75-8.87	0.118
	Ignored	0	0	8	100	8	100	-	-	0.215
	No	61	16.62	306	83.38	367	100	Ref	-	-
Meningitism	Yes	0	0	3	100	3	100	-	-	0.44
	Ignored	1	12.5	7	87.5	8	100	0.72	0.09-5.93	0.756
	No	44	15.28	244	84.72	288	100	Ref	-	-
Other symptoms	Yes	10	13.7	63	86.3	73	100	0.88	0.42-1.85	0.735
	Ignored	8	47.06	9	52.94	17	100	4.93	1.8-13.47	<0.001
	No	20	9.66	187	90.34	207	100	Ref	-	-
**Hospitalization**	Yes	41	24.85	124	75.15	165	100	3.09	1.73-5.53	<0.001
	Ignored	1	16.67	5	83.33	6	100	1.87	0.2-16-8	0.57
	Clinical-epidemiological	4	14.29	24	85.71	28	100	Ref	-	-
Diagnosis criteria	Clinical-laboratory	58	16.86	286	83.14	344	100	1.22	0.4-3.64	0.725
	No Filling	0	0	6	100	6	100	-	-	0.324
Autochthonous **	No	13	81.25	3	18.75	16	100	Ref	-	-

	Yes	34	61.82	21	38.18	55	100	0.37	0.1-1.47	0.148
	Indeterminate	15	71.43	6	28.57	21	100	0.58	0.12-2.78	0.49
	No Filling	0	0	286	100	286	100	-	-	<0.001
Evolution	Healing	50	14.62	292	85.38	342	100	Ref	-	-
	Ignored	2	15.38	11	84.62	13	100	1.06	0.23-4.93	0.939
	Death other causes	1	7.14	13	92.86	14	100	0.45	0.06-3.51	0.434
	Death by leptospirosis	9	100	0	0	9	100	-	-	<0.001

* OR cannot be computed because it has zero ** Variable not included in multiple high hair modeling Number of “No Filling”

Table S2. Multiple logistic regression models by the stepwise method of input and output of Human variables.

**Variáveis**	**Model 1**	**Model 2**	**Model 3**	**Model 4**	**Model 5**	**Model 6**	**Model 7**	**Model 8**	**Model 9**	**Model 10**	**Model 11**
	***p*-value**	***p*-value**	***p*-value**	***p*-value**	***p*-value**	***p*-value**	***p*-value**	***p*-value**	***p*-value**	***p*-value**	***p*-value**
Intercept	<0.001	<0.001	<0.001	<0.001	<0.001	<0.001	<0.001	<0.001	<0.001	<0.001	<0.001
**Sex: Male**	0.088	0.060	0.059	0.055	0.053	0.048	0.052	0.067	0.056	0.044	0.042
Illiterate	0.997										
Education: High school	0.268										
Education: Higher education	0.831										
Education Not applicable	0.994										
Education No Filling	0.282										
Risk Flood water or mud: No Filling	0.992	0.991	0.991	0.991	0.991	0.991	0.990	0.990	0.990	0.990	0.989
Risk Flood water or mud: yes	0.299	0.185	0.180	0.185	0.195	0.191	0.208	0.194	0.139	0.105	0.102
Risk River, stream, pond or dam: yes	0.201	0.162	0.159	0.159	0.159	0.134	0.184	0.168			
Risk River, stream, pond or dam: No Filling	0.624	0.663	0.665	0.668	0.704	0.896	0.771	0.687			
Risk Wasteland: yes	0.613	0.685	0.681	0.671	0.690	0.687	0.551	0.605	0.798	0.850	
Risk Wasteland: No Filling	0.342	0.339	0.338	0.342	0.334	0.310	0.229	0.179	0.086	0.089	
Risk Garbage/debris: yes	0.713	0.701	0.703	0.688	0.656	0.676	0.572	0.668	0.642	0.676	0.587
Risk Garbage/debris: No Filling	0.991	0.991	0.990	0.990	0.990	0.990	0.990	0.990	0.989	0.989	0.989

**Fever: yes**	0.093	0.095	0.094	0.094	0.090	0.083	0.100	0.099	0.099		
Fever: Ignored	0.999	0.999	0.999	0.999	0.996	0.996	0.995	0.995	0.995		
Myalgia: yes	0.318	0.278	0.263	0.265	0.239	0.220	0.207	0.177	0.174	0.063	0.075
Myalgia: Ignored	0.997	0.997	0.996	0.996	0.995	0.995	0.994	0.994	0.994	0.993	0.992
Conjunctival congestion: yes	0.120	0.111	0.112	0.105	0.097	0.125	0.232				
Conjunctival congestion: Ignored	0.999	0.999	0.999	0.999	0.999	0.999	0.999				
**Calf pain: yes**	0.008	0.007	0.007	0.007	0.007	0.006	0.009	0.008	0.006	0.007	0.005
Calf pain: Ignored	0.995	0.995	0.994	0.994	0.994	0.994	0.994	0.994	0.993	0.993	0.993
Vomit: yes	0.792	0.726	0.688	0.688							
Vomit : Ignored	0.998	0.998	0.996	0.996							
Diarrhea : yes	0.917	0.865									
Diarrhea: Ignored	0.997	0.997									
**Jaundice: yes**	<0.001	<0.001	<0.001	<0.001	<0.001	<0.001	<0.001	<0.001	<0.001	<0.001	<0.001
Jaundice: Ignored	0.997	0.997	1.000	0.999	0.999	0.999	0.998	0.998	0.998	0.997	0.997
**Renal insufficiency: yes**	0.025	0.017	0.017	0.017	0.016	0.014	0.014	0.009	0.008	0.004	0.008
Renal insufficiency: Ignored	0.998	0.998	0.998	0.998	0.998	0.998	0.998	0.997	0.997	0.997	0.997
Respiratory alteration: yes	0.203	0.210	0.210	0.198	0.210	0.237					
Respiratory alteration: Ignored	0.996	0.996	1	1	1	1					
Pulmonary hemorrhage: yes	0.974	0.827	0.837								
Pulmonary hemorrhage: Ignored	0.998	0.998	0.998								
Other hemorrhages: yes	0.994	0.993	0.993	0.993	0.993	0.993		0.993	0.993	0.993	0.993
Other hemorrhages: Ignored	1	1	1	1	1	1		1	1	1	1
Hospitalization: yes	0.550	0.501	0.515	0.512	0.514		0.993				
Hospitalization: Ignored	0.564	0.540	0.542	0.541	0.551		1.000				
Evolution: Death other causes	0.544	0.685	0.672	0.610	0.594	0.659	0.661	0.575	0.536	0.472	0.503
Evolution: Death by leptospirosis	0.989	0.989	0.988	0.988	0.988	0.988	0.987	0.987	0.987	0.987	0.986
Evolution: Ignored	0.666	0.797	0.793	0.792	0.795	0.780	0.804	0.865	0.934	0.812	0.784

* OR no pode ser calculada por possuir zero

Table S3. Number of co-variables from Human Models and their respective AIC.

Model	Number de co-variables	AIC
Model 1	41	283.52
Model 2	36	277.87
Model 3	34	273.90
Model 4	32	269.96
Model 5	30	266.13
Model 6	28	263.11
Model 7	26	260.61
Model 8	24	257.98
Model 9	22	256.33
Model 10	20	255.38
Model 11	18	255.28

Table S4. Associated risk factors for anti-*Leptospira* agglutinins (2019-2020) in dog population of of Pinhais. Paraná state. southern Brazil (N = 133). by uni- and multivariate statistical analysis.

Variable		Positive		Negative		*p*-value*	OR	IC	*P*-value
		N	lin %	N	lin %				
Animal sex	Female	5	6.41	73	93.59	0.808	0.685	0.188-2.49	0.564
	Male	5	9.09	50	90.91		Ref	-	-
Age range	Up to 1 year	1	8.33	11	91.67	0.961	Ref	-	-
	1 to 8 years	7	7.53	86	92.47		0.895	0.100 -7.978	0.921
	Above 8 years	2	8.70	21	91.30		1.048	0.085-12.876	0.971
Classification	Domiciled	9	7.83	106	92.17	1	Ref	-	-
	Wandering + Semi-Domiciled	1	5.56	17	94.44		0.693	0.082-5.821	0.734
Ethinicity	One ethinicity	0	0.00	33	100.00		0.000	-	0.056
	Mixed	1	10.20	88	89.80	0.126	Ref	-	-
Body score	1 (very thin)	0	0.00	1	100.00		0.000		0.784

	2 (thin)	0	0.00	9	100.00	0.429	0.000		0.412
	3 (normal)	4	7.02	53	92.98		Ref	-	-
	4 (fat)	2	4.88	39	95.12		0.679	0.118- 3.899	0.663
	5 (very fat)	4	16.00	21	84.00		2.524	0.577-11.034	0.207
	Big	1	3.45	28	96.55		0.690	0.069 -6.940	0.752
Size	Average	6	13.95	37	86.05	0.146	3.135	0.738-13.311	0.106
	Small	3	4.92	58	95.08		Ref	-	-
Castrated	No	7	8.33	77	91.67		Ref	-	-
	Yes	3	6.12	46	93.88	0.9	0.717	0.177-2.912	0.641
Dog tick collection	No (ref)	9	11.84	67	88.16	0.064	Ref	-	-
	Yes	1	1.75	56	98.25		0.133	0.016-1.082	0.029
Number of ticks collected from dogs	0	10	12.35	71	87.65	0.074			
	1 to 5	0	0	36	100				
	6 to 10	0	0	4	100				
	More than 10	0	0	12	100				
Environmental tick collection	No (ref)	3	4.76	60	95.24	0.415	Ref	-	-
	Yes	7	10	63	90		2.222	0.549-8.993	0.253
Number of ticks collected from the environment	0 (ref)	3	4.76	60	95.24	0.123	Ref	-	-
	1 to 5	5	14.29	30	85.71		3.333	0.746-14.893	0.099
	6 to 10	0	0	5	100		0.000		0.618
	11 to 20	2	20	8	80		5.000	0.722-34.631	0.076
	More than 20	0	0	20	100		0.000		0.320
Tick collection sites (OR environment X no collection)	Environmental	7	21.88	25	78.12	0.003	Ref	-	-
	No tick collection	3	6	47	94		0.228	0.054-0.959	0.032
Dog housing location	Inside home	0	0	3	100	0.810			
	Backyard	10	7.81	118	92.19				
	Street	0	0	2	100				
	1	2	11.76	15	88.24	0.764	Ref	-	-

Number of dogs	2	3	7.69	36	92.31		0.625	0.095-4.128	0.623
	3 or more	3	6.25	45	93.76		0.500	0.076-3.284	0.463
Other animals	No	7	11.48	54	88.52	0.207	2.981	0.736-12.074	0.111
	Yes	3	4.17	69	95.83		Ref	-	-
Animal mobility	Street access	2	7.69	24	92.31	0.611	Ref	-	-
	Dog kennel	0	0	11	100		0		0.344
	Released in the backyard	8	8.33	88	91.67		1.091	0.217-5.479	0.916
Enter the house	No	2	2.99	65	97.01	0.095	Ref	-	-
	Yes	8	12.12	58	87.88		4.483	0.915-21.969	0.046
Access to forest	No	7	7.37	88	92.63	1	Ref	-	-
	Yes	3	7.89	35	92.11		1.078	0.264-4.405	0.917
Visualization of rodents	No	2	3.45	56	96.55	0.217	Ref	-	-
	Yes	8	10.67	67	89.33		3.343	0.682-16.388	0.117
Rodent viewing period	Day	2	14.29	12	85.71	0.345	Ref	-	-
	Night	3	7.89	35	92.11		0.514	0.076-3.458	0.488
	Both	3	13.04	20	86.96		0.9	0.131-6.182	0.915
	Does not visualize	2	3.45	56	96.55		0.214	0.027-1.676	0.112
Food	Food	1	20	4	80	0.558	3.250	0.303-34.857	0.306
	Portion	5	7.14	65	92.86		Ref	-	-
	Portion and Food	4	6.9	54	93.1		0.963	0.246-3.765	0.957
Carne cStreet	No	9	8.04	103	91.96	0.943	Ref	-	-
	Yes	1	4.76	20	95.24		0.572	0.069-4.771	0.602
Water	Supply network/Free access	10	7.52	123	92.48				
Bleeding	No	9	7.56	110	92.44	1	Ref	-	-
	Yes	1	7.14	13	92.86		0.940	0.110-8.027	0.955
Local	Anus	0	0	1	100	0.723			
	Mouth	0	0	2	100				
	Head	0	0	1	100				

	Back	0	0	1	100				
	Feces	0	0	3	100				
	Genital	1	50	1	50				
	Not informed	0	0	2	100				
	There was no bleeding	9	7.56	110	92.44				
	Ear	0	0	1	100				
	Left hind paw	0	0	1	100				
Vomit	No	7	6.67	98	93.33	0.75	Ref	-	-
	Yes	3	10.71	25	89.29		1.68	0.405-6.964	0.470
Diarrhea	No	10	8.06	114	91.94	0.817	Ref	-	-
	Yes	0	0	9	100		0.000	-	0.376
Slimming	No	10	8.13	113	91.87	0.753	Ref	-	-
	Yes	0	0	10	100		0	-	0.348
Tick season	Whole year	2	12.50	14	87.50	0.392	1	0.123-8.128	1
	Don’t know	1	20.00	4	80.00		1.75	0.124-24.650	0.676
	Autumn + Winter	2	12.50	14	87.50		Ref	-	-
	Spring + Summer	5	5.21	91	94.79		0.385	0.068-2.177	0.265
Control Ticks	No	1	2.94	33	97.06	0.426	Ref	-	-
	Yes	9	9.09	90	90.91		3.3	0.402-27.060	0.241
Presence of fleas	No	5	6.25	75	93.75	0.729	Ref	-	-
	Yes	5	9.43	48	90.57		1.563	0.429-5.684	0.495
Flea control	No	8	8.25	89	91.75	0.743	Ref	-	-
	Yes	2	6.67	28	93.33		0	-	0.464
	Don’t know	0	0	6	100		0.795	0.159-3.962	0.779
Vaccination	Both	4	7.14	52	92.86	0.751	0.577	0.134-2.477	0.455
	Anti-rabies	2	6.45	29	93.55		0.517	0.088-3.044	0.460
	Polyvalent	0	0	6	100		0	-	0.376
	None	4	11.76	30	88.24		Ref	-	-

	Don't know	0	0	6	100		0	-	0.376
	No	6	7.89	70	92.11	0.947	Ref	-	-
Vaccination Annual	Yes	4	7.14	52	92.86		0.897	0.241-3.343	0.872
	Don't know	0	0	1	100		0.000	-	0.770
	No	3	7.89	35	92.11	0.956	Ref	-	-
Deworming	Yes	7	7.45	87	92.55		0.939	0.230-3.838	0.930
	Don't know	0	0	1	100		0	-	0.770
	1 time	1	3.23	30	96.77	0.696	-	-	0.630
	2 times	1	25	3	75		-	-	0.165
	3 times	0	0	3	100		-	-	
Frequency	Yearly	3	8.33	33	91.67		-	-	0.428
	Never	3	10.71	25	89.29		-	-	0.365
	No	0	0	7	100		Ref	-	-
	Don't know	2	8.33	22	91.67		-	-	0.430
Animal Hygiene	Clean	10	11.9	74	88.1	0.030	Ref	-	-
	Dirty	0	0	49	100		0	-	0.012

* Chi Square Test

Table S5. Multiple logistic regression models by the stepwise method of input and output of dog variables.

**Variable**	**Model 1**	**Model 2**	**Model 3**	**Model 4**	**Model 5**
	***p*-value**	***p*-value**	***p*-value**	***p*-value**	***p*-value**
Intercept)	0.027	0.027	0.014	0.005	0.003
Size: Average	0.248	0.253			
Size: Small	0.936	0.959			
Tick collection on the dog: yes	0.853				
Tick collection sites: Both	0.994	0.994	0.995	0.995	0.995
Tick collection sites: Dog	0.997	0.997	0.997	0.997	0.996

No tick collection	0.105	0.107	0.049	0.034	0.021
Other animals: yes	0.264	0.270	0.350		
Enter the house: yes	0.077	0.078	0.077	0.070	0.059
Visualization of rodents: yes	0.295	0.300	0.313	0.292	
